# Factors associated with postoperative outcomes in patients with intramedullary Grade II ependymomas: A Systematic review and meta-analysis

**DOI:** 10.1097/MD.0000000000016185

**Published:** 2019-06-21

**Authors:** Xiang-Yao Sun, Wei Wang, Tong-Tong Zhang, Chao Kong, Si-Yuan Sun, Ma-Chao Guo, Jun-Zhe Ding, Shi-Bao Lu

**Affiliations:** a Department of Orthopedics, Xuanwu Hospital Capital Medical University; b National Clinical Research Center for Geriatric Diseases; c Capital Medical University; d Department of Orthopaedics, ChuiYangLiu Hospital Affiliated to Tsinghua University, Beijing, China.

**Keywords:** associated factors, individual patient data analysis, intramedullary ependymomas, overall survival, progression-free survival

## Abstract

**Background:**

Most of the previous studies combined all types of intramedullary ependymomas without providing accurate pathological subtypes. In addition, it was very difficult to evaluate the factors associated with postoperative outcomes of patients with different pathological subtypes of intramedullary Grade II ependymomas by traditional meta-analysis. This study evaluated the factors related with postoperative outcomes of patients with intramedullary Grade II ependymomas.

**Methods:**

Individual patient data analysis was performed using PubMed, Embase, and the Cochrane Central Register of Controlled Trials. The search included articles published up to April 2018 with no lower date limit on the search results. The topics were intramedullary Grade II ependymomas. Progression-free survival (PFS) and overall survival (OS) were analyzed by Kaplan–Meier survival analysis (log-rank test). The level of significance was set at *P *<* *.05.

**Results:**

A total of 21 studies with 70 patients were included in this article. PFS of patients who underwent total resection was much longer than the PFS of those who received subtotal resection (*P* < .001). Patients who received adjuvant therapy (*P* = .005) or radiotherapy and chemotherapy (*P* < .001) seemed to have shorter PFS than others; PFS of patients who had cerebrospinal fluid disease dissemination (*P* = .022) or scoliosis (*P* = .001) were significantly shorter than others. OS of cellular ependymoma patients was less than giant cell ependymoma patients (*P* < .001).

**Conclusions:**

PFS of patients who received total resection was much longer than those who received subtotal resection. Patients treated with adjuvant therapy or radiotherapy and chemotherapy appeared to have shorter PFS than others; PFS of patients with cerebrospinal fluid disease dissemination or scoliosis were significantly shorter than others. Cellular ependymomas would have better OS than giant cell ependymoma. However, giant cell ependymoma patients might have the worst OS.

## 1 Introduction

In adults, ependymomas are the most common tumors of the spinal cord, accounting for 60% of tumors in this region.^[1]^ Based on the degree of microscopic malignancy, ependymomas are classified into 3 grades according to the World Health Organization (WHO) histologic subtypes.^[2]^ Grade I lesions, which include myxopapillary ependymoma and subependymoma, are the most benign in histologic appearance. Grade II ependymomas, which are also known as “classic ependymomas,” account for about 55% to 75% of tumors in the spinal cord. In addition, Grade II ependymomas are most commonly seen in the cervical or thoracic cord and relatively rare in the lumbar region. Grade III lesions are anaplastic ependymomas, which correspondingly have the most malignant behavior.^[3]^ Characteristic histologic features of Grade II ependymomas are pseudorosettes (in 80% of ependymal tumors) and “true” or “ependymal” rosettes (10% of ependymal tumors); the other 10% of ependymal tumors lack characteristic histologic features.^[4,5]^ This subtype of ependymomas usually has well-defined margins with compression of the adjacent tissue.^[3]^

WHO classifies Grade II ependymomas into several subtypes: cellular ependymomas, which are described as having hypercellularity, a high nuclear-to-cytoplasm ratio, and few rosettes, but lack mitoses, cellular pleomorphism, and microvascular proliferation; clear cell ependymomas, which are relatively rare, are identified by perinuclear halos, sharp histologic borders, and pseudorosettes; papillary ependymomas contain neoplastic cells around a fibrovascular core; tanycytic ependymomas, which are more common in the spinal cord than the brain, are identified by cells with long processes.^[2]^ Giant cell ependymomas are relatively rare subtypes of Grade II ependymomas, characterized by pleomorphic giant cells.^[6]^

In this study, we extracted data from case reports on patients with different pathological subtypes of intramedullary Grade II ependymomas, and performed an individual patient data analysis using the methods of survival analysis in order to ensure reliability of the results.

## 2 Materials and methods

### 2.1 Search strategy

The primary sources for the literature review were PubMed, Embase, and the Cochrane Central Register of Controlled Trials, to identify trials according to Cochrane Collaboration guidelines. The search included articles published from January 1980 up to April 2018 with no lower date limit on search results. In order to verify the accuracy of Sun et al's study,^[7]^ we followed their methods. The following search terms and different combinations of Medical Subject Heading terms and textual words were used: “spinal cord ependymoma,” “intramedullary ependymoma,” “papillary ependymoma,” “cellular ependymoma,” “clear cell ependymoma,” “giant cell ependymoma,” “tanycytic ependymoma,” “exophytic ependymoma,” and “spinal cord neoplasm.” Manual searches of the reference lists of all included studies were used to identify studies that the electronic search may have failed to identify.

### 2.2 Selection criteria

We included studies where:

(1) the information for a single case was presented;
(2) case reports, case series reviews, or observational studies were performed;
(3) the subtype of Grade II ependymoma according to the WHO was clearly given for the diagnosis^[2]^;
(4) complications, events of recurrence, death, follow-up period, and other factors relevant to the postoperative outcomes of the disease were provided in the articles;
(5) the language of studies was limited to English.

However, exclusion criteria for this study consisted of the following:

(1) the study failed to focus on Grade II ependymoma;
(2) patient data was not available;
(3) follow-up data was not referenced;
(4) the primary lesion was extramedullary;
(5) patients had other tumors and severe diseases, such as neurofibromatosis and malignant tumor metastasis;
(6) the full text of the article was unavailable.

### 2.3 Data extraction

Two investigators independently extracted data from included studies. Information regarding patient demographics, inclusion and exclusion criteria, interventions, outcomes, pathology of tumor, location and length of tumor, complications, and follow-up time was extracted. Patients were divided into groups according to possible influencing factors. Patients were divided into 2 age groups (age <18 and age ≥18) and 2 groups according to gender (male and female). Two groups (≥3 vertebra levels and < 3 vertebra levels) were made according to tumor length. Patients were divided into 5 groups according to pathologic classifications: cellular ependymoma, papillary ependymoma, clear cell ependymoma, tanycytic ependymoma, and giant cell ependymoma. Total resection (TR) was defined as the tumor being removed piecemeal or in an en bloc fashion. Therefore, 3 groups were categorized according to the extent of resection: TR, subtotal resection (STR), and biopsy and decompression. Patients were divided into groups (Done and Not) according to whether adjuvant therapies were applied or not. Adjuvant therapies included radiotherapy (RT) only and RT and chemotherapy, considering that chemotherapy only was not applied in the included studies. RT was categorized into 2 groups (RT only and RT and chemotherapy) according to whether chemotherapies were applied or not. The clinical outcomes of patients included tumor recurrence, death, and other complications. Patients were analyzed according to different kinds of complications: tumor disseminated by cerebrospinal fluid (CSF), CSF leak, wound dehiscence, infection, pseudomeningocele, and scoliosis. Progression of tumor was defined as recurrence, tumor-relevant death, and repeat surgery. Death due to causes other than the intramedullary Grade II ependymomas were represented as censored data in this research. All the grouping standards were determined by clinical experiences or literatures. Missing information was listed as censored data in the analysis. Data from duplicated cases were consolidated.

### 2.4 Quality of the studies

The quality of the studies was assessed according to the level of evidence^[8]^: Level I, which includes randomized controlled trial (RCT) and meta-analysis of randomized trials with homogeneous results; Level II, which includes poorly designed RCT, prospective cohort study (therapeutic) and meta-analysis of Level II studies; Level III, which includes retrospective cohort study, case–control study and meta-analysis of Level 3 studies; Level IV, which includes case series; Level V, which includes case report, expert opinion and personal observation. Two reviewers (XYS and SYS) independently assessed the titles and abstracts and excluded duplicate results, and then screened studies according to inclusion and exclusion criteria. Full-text articles were retrieved when inclusion was unclear, according to abstracts. Studies that failed to meet the inclusion criteria were excluded. Any disagreements were resolved by a third researcher (SBL).

### 2.5 Statistical analyses

Statistical analysis was performed using the Statistical Package for Social Sciences version 17.0 software (SPSS, Inc, Chicago, IL). Continuous data were shown as the mean ± standard deviation. Dichotomous data were reported as the number or ratio. The Kolmogorov–Smirnov test was used to analyze normality of continuous data. One-way analysis of variance or the Student *t* test was used to analyze normally distributed values. The Kruskal–Wallis test was used to analyze skew distributed values. Pearson chi-squared test was performed to discuss the statistical significance of noncontiguous data. Progression-free survival (PFS) and overall survival (OS) were analyzed by Kaplan–Meier survival analysis (log-rank test). The level of significance was set at *P* < .05.

## 3 Results

### 3.1 Study characteristics

**Figure 1:**
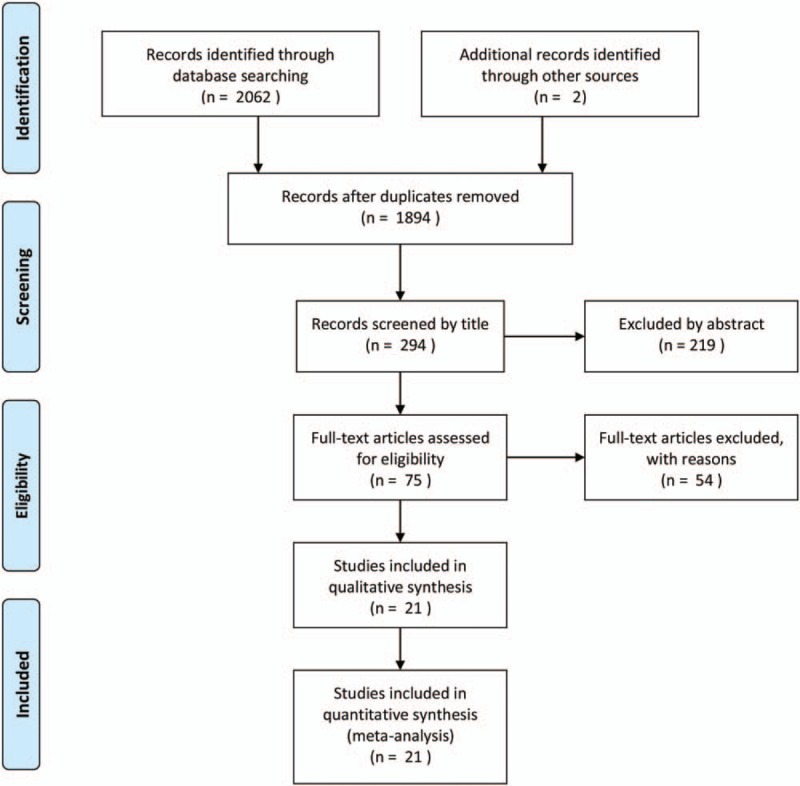
Flowchart showing results of the study search.

Figure 1 shows a flow chart of the study selection and inclusion process. The search strategy identified 2062 potential articles, of which 21 articles^[3,9–28]^ satisfied predefined inclusion criteria for data extraction and statistical analysis. In all these articles,^[6,9,11,14,19,22,25]^ were nonrandomized trials and observational studies (Level III) and 15^[3,10,12,13,15–18,20,21,23,24,26–28]^ were case studies (Level IV).

### 3.2 Demographic features

Some variables appeared in several references, for example, sex, while others, for example, scoliosis, appeared a fewer references (Table 1). A total of 70 patients were evaluated. The mean age was 40.5 ± 15.6 years. There was a balanced sex ratio (male, 48.6%). Tumors were most likely to be located in thoracic vertebral levels (41.4%), while they were less likely to occur in thoracolumbar vertebral levels (1.4%). More than half of the patients’ tumor length was more than 3 vertebral levels (59.3%). Cellular ependymoma patients seemed to be the most common subtype of Grade II ependymomas in the included studies with an incidence rate of 65.7%. Most of the patients received TR (65.7%). Adjuvant therapies reported in these articles included RT only (34.3%) and RT combined with chemotherapy (8.6%). Chemotherapy alone was not applied in these studies. Diffuse infiltration of the tumor (15.7%) appeared to be the most common complication in these studies. The recurrence rates at 1 and 5 years were 11.4% and 30%, respectively.

**Table 1:**
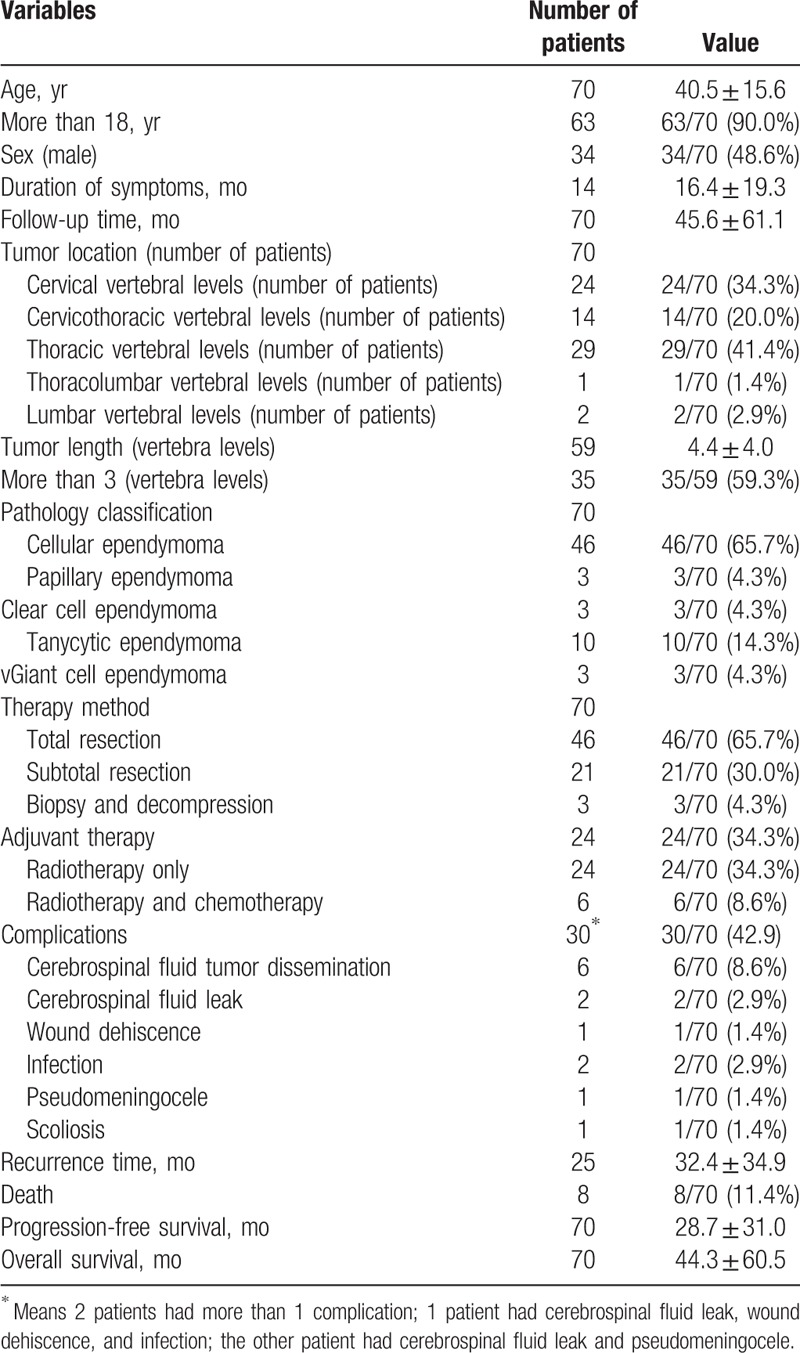
Summary of data in the included studies.

### 3.3 Progression-free survival

A total of 25 (35.7%) patients experienced tumor recurrence (Supplementary material 1). Recurrence time was 32.4 ± 34.9 months. Results of the Kaplan–Meier analysis showed that PFS was not associated with patient age (*P* = .387), sex (*P* = .734), tumor length (*P* = .880), treatment methods (*P* = .631), or CSF leak (*P* = .631) (Table 2). In addition, tumor location (*P* = .305) and pathological classification (*P* = .359), wound dehiscence (*P* = .757), infection (*P* = .589), and pseudomeningocele (*P* = .716) were not associated with PFS. PFS of patients who received TR was much longer than those who received STR (*P* < .001). There was no significant difference between TR and biopsy and decompression (*P* = .793). No significant difference was found between STR and biopsy and decompression (*P* = .060). Patients who received adjuvant therapy (*P* = .005) or RT and chemotherapy (*P* < .001) appeared to have shorter PFS than others. PFS of patients who had CSF disease dissemination (*P* = .022) or scoliosis (*P* = .001) were significantly shorter than others (Table 1). Symptomatic duration was not associated with the recurrence of tumors (*P* = .269).

**Table 2:**
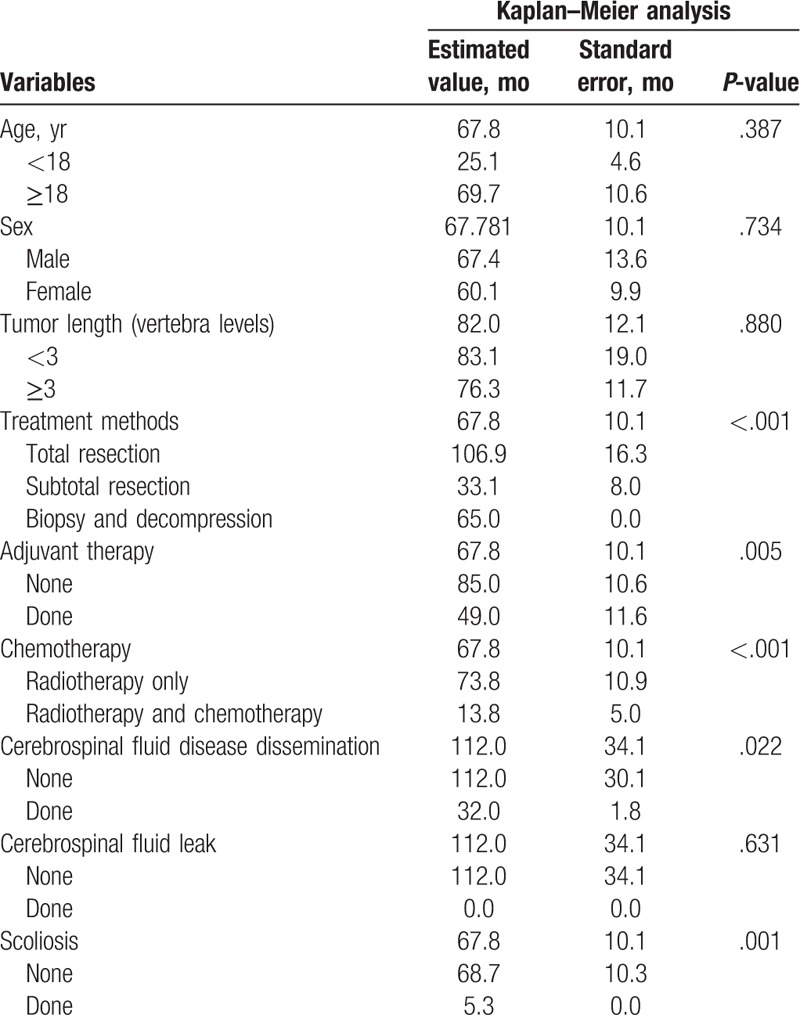
Kaplan–Meier analysis of progression-free survival in intramedullary Grade II ependymoma patients.

### 3.4 Overall survival

**Figure 2:**
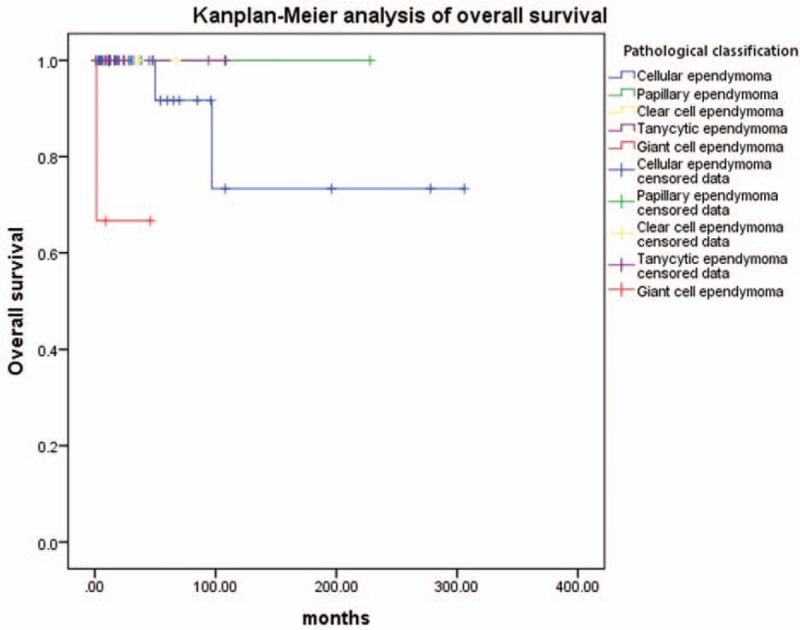
Kaplan–Meier analysis of overall survival in patients with different pathological classifications.

The OS analysis was not performed in relationship to cervical vertebra levels, cervicothoracic vertebra levels, thoracic vertebra levels, TR, STR, biopsy and decompression, RT only, RT and chemotherapy, CSF disease dissemination, CSF leak, wound dehiscence, infection, pseudomeningocele, or scoliosis, due to the unavailability of survival data. The Kaplan–Meier analysis showed that age (*P* = .387), sex (*P* = .734), tumor location (*P* = .305), tumor length (*P* = .880), treatment methods (*P* = .135), adjuvant therapy (*P* = .937), CSF disease dissemination (*P* = .617), and diffuse infiltration (*P* = .114) did not influence OS (Table 2). There was no significant difference in OS between intramedullary Grade II ependymoma patients with recurrence and those without recurrence (*P* = .317). In the total population, 7 patients (10.0%) died of cellular ependymomas; 1 patient (1.4%) died of giant cell ependymomas; there were no patients with other kinds of intramedullary Grade II ependymomas who died. In the cellular subtype, the morality rate of cellular ependymomas and giant cell ependymomas were 15.2% and 33.3%, respectively. Therefore, OS could not be calculated in patients with papillary ependymomas, clear cell ependymomas, or tanycytic ependymomas. There was a significant difference among different subtypes of intramedullary Grade II ependymomas (*P* < .001). The OS of cellular ependymoma patients was better than that of giant cell ependymoma patients (*P* < .001) (Table 3 and Fig. 2).

**Table 3:**
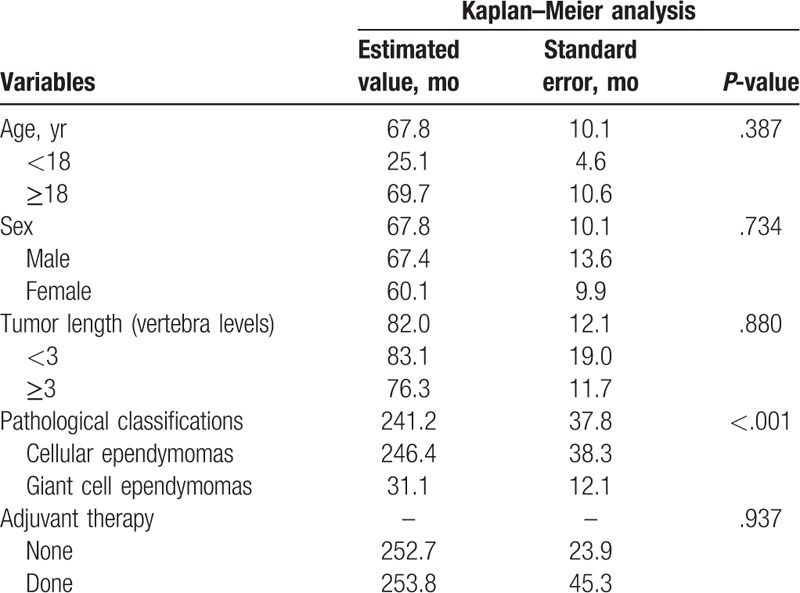
Kaplan–Meier analysis of overall survival in intramedullary Grade II ependymoma patients.

## 4 Discussion

Previous studies have discussed the influencing factors for surgical outcomes of patients with ependymomas; they showed that TR was associated with better PFS or OS even after controlling for adjuvant radiation therapy.^[3,7]^ However, there are a limited number of articles focusing on Grade II ependymomas. Several studies suggested that Grade II ependymoma patients with TR, compared to patients with STR, had a significantly lower recurrence rate; there is also a significant association between tumor histology and extent of resection, with a higher rate of TR in Grade II ependymoma patients; thoracic level Grade II ependymoma patients usually had poor progression.^[3,7,8]^ If full resection of the tumor is not possible, adjunctive RT is recommended; however, RT may improve PFS regardless of the extent of resection.^[9]^ The role of chemotherapy for Grade II ependymomas is less clear than for RT. Most of the studies are case reports, which lack statistical analysis. In addition, the relationship between complications and clinical outcomes in Grade II ependymoma patients has not been fully discussed in previous studies.^[3,7,8]^ Therefore, there is still an urgent need for further investigation of postoperative outcomes of Grade II ependymomas. Several studies^[10–12]^ analyzed postoperative outcomes and recurrence in cases of spinal cord and cauda equina ependymoma; however, subtypes of Grade II ependymomas were not discussed separately, considering the limited cases in these studies as data to be analyzed included Grade I to III ependymomas, which made the results inaccurate.

Considering that most of the studies combined all types of intramedullary ependymomas without providing accurate pathological subtypes, evaluating the factors that were associated with postoperative outcomes of patients with different pathological subtypes of intramedullary Grade II ependymomas, by traditional meta-analysis, was impossible. Even though Sun et al^[13]^ discussed the differences of PFS and OS between different subtypes of Grade II ependymomas, their study included many typical Grade II ependymoma patents without explicit pathological subtypes, which would make the conclusions less persuasive; in addition, perioperative complications were not fully discussed in their research, which would make the evaluative systems of clinical outcomes incomplete. There is an urgent need to verify the correctness of their conclusions. Therefore, we evaluated the factors related with postoperative outcomes of patients with intramedullary Grade II ependymomas in this study.

Previous studies stated that predictors of poor clinical behavior in intramedullary ependymoma patients included age less than 3 years, p53 immunopositivity, a high proliferation index, and incomplete resection.^[29–31]^ It has been reported that younger age was associated with improved long-term survival.^[32]^ Alshaya et al^[33]^ stated that younger age was the only risk factor for patients with TR of the lesion. However, none of these studies had large sample size or focused on intramedullary Grade II ependymomas. In this analysis, age, sex, tumor length, or tumor location was not associated with PFS or OS; this may be partly explained by the same WHO classification of intramedullary Grade II ependymomas in this research.

### 4.1 The grade of ependymomas

Previous studies reported that tumor grade was significantly associated with survival; however, PFS did not differ across histologic grades.^[34,35]^ Though the prognostic importance of histologic features is controversial, compared with grade III, most large studies show better PFS and OS for grade I or grade II histology.^[36]^ In this article, no significance in PFS is found among different subtypes of intramedullary Grade II ependymomas; no patients with intramedullary Grade II ependymomas died, except patients with cellular ependymomas or giant cell ependymomas. Hypercellularity and a high nuclear-to-cytoplasm ratio with few rosettes of cellular ependymomas manifested its relatively high proliferative potential combined with low cure rate.^[3]^ If giant cells in giant cell ependymomas can be mistaken for gemistocytes in the absence of perivascular pseudorosette formation, they may be treated with wrong therapeutic strategy.^[21]^ Therefore, cellular ependymomas and giant cell ependymomas may have worse OS than other subtypes. However, Sun et al^[7]^ stated that there was no difference in OS between cellular ependymomas and other subtypes. This is because their study included many patients with typical Grade II ependymoma, which could be classified into pathological subtypes. All these patients would cause bias in the results.

### 4.2 Excision extension

Previous studies stated that complete resection was associated with improvement in survival of spinal cord ependymomas.^[35,37,38]^ This study found that PFS significantly improved in the TR group. Similarly, Prokopienko et al^[39]^ stated that TR is an ideal treatment of choice in intramedullary ependymomas; no tumor recurrence occurred after TR. Kobayashi et al^[40]^ analyzed postoperative outcomes and recurrence in patients with spinal cord and cauda equina ependymoma; they found that in cases undergone TR, the recurrence rate was significantly reduced. Their results also revealed that a good preoperative motor status also caused significantly better postoperative recovery of motor status and they recommended early surgery for spinal cord and cauda equina ependymoma before aggravation of paralysis. These results imply that residual tumor may be associated with PFS. STR may be used when TR is difficult. It was reported that those who did not achieve complete resection could still have maximal safe resection with postoperative RT.^[41]^ Previous study showed that there was an association between extent of resection and PFS in patients with spinal cord ependymoma; however, evidence of definitive relationships with PFS or OS were lacking due to the rarity of intramedullary Grade II ependymomas; indeed, not all patients might benefit from more complete resection.^[1]^

### 4.3 Adjuvant therapy

The role of adjuvant radiation therapy in the treatment of Grade II ependymomas remains controversial. The option of observation following en bloc complete resection for patients with low-grade spinal cord ependymomas (myxopapillary ependymoma and subependymoma) is a reasonable one. If the tumor persists on the postoperative MRI, RT is recommended.^[40]^ It has been reported that incomplete resection for patients with postoperative RT had excellent local control and survival.^[41]^ RT typically includes fractionated external beam therapy to a cumulative dose of 54 Gy, which has been shown to control the local tumor.^[3]^ However, Amirian et al^[42]^ stated that RT in adults was detrimental to survival. This study finds patients who are treated with adjuvant therapy to have a higher risk of progression than those without adjuvant therapy; however, no significant difference was found in OS between patients who received adjuvant therapy and those who did not. Chronic oral etoposide is widely used in the chemotherapy of Grade II ependymomas, because of its novel mechanism of action, ease of administration and modest toxicity.^[11]^ It has been reported that the use of chemotherapy before RT should depend on the age of the patient; given the lack of evidence that chemotherapy is effective against tumor both in the brain and spine, the role of chemotherapy is limited; when chemotherapy is employed, its duration is limited and local control measures, such as surgery and RT, should be performed early.^[43]^ In this study, patients who received RT and chemotherapy have worse PSF than others. This might be explained by the fact that patients who received adjuvant therapy had tumor with a higher grade of malignancy compared to others. Routine postoperative RT is not indicated after TR.^[43]^ Postoperative adjuvant therapy, in general, depends on the extent of resection and symptomatology. It seems that PFS is associated with the residual tumor tissue. Most of the patients would receive adjuvant therapy as salvage therapy for recurrence if the surgery failed.^[3]^ Such instances would have an adverse impact on the efficacy analysis of adjuvant therapy. Because there was no significant difference in OS between patients who received adjuvant therapy and those who did not receive adjuvant therapy. Adjuvant therapy may improve the prognosis of patients with worse baseline and achieve the same OS as others. Considering that an adequate RT or chemotherapy dose to the tumor and margins are necessary, if doubt exists as to the local or disseminated nature of the tumor at the time of applying adjuvant therapies, CSF cytology and spinal MR imaging should be used to make the final decision.^[43]^

### 4.4 Complications

Different kinds of complications were found in our study: tumor disseminated by CSF, CSF leak, wound dehiscence, infection, pseudomeningocele, and scoliosis. A possible explanation for subarachnoid dissemination includes intercellular adhesion; cellular shedding in the surgical specimen could be observed in surgical specimens of cases with subarachnoid dissemination; zonula adherens junctions were poorly developed and infrequently identified, even though zonula occludens junctions were abundant, possibly accounting for the decreased intercellular adhesion.^[12]^ Another factor possibly facilitating dissemination could be repeat resections, which free tumor cells into the CSF, as sometimes can occur in choroid plexus papillomas^[44]^ and pituitary adenomas.^[43]^ CSF dissemination or distant metastasis predicts a poor prognosis.^[45]^ Although, Connolly et al^[46]^ stated that anatomic sequestration and the low grade of intramedullary spinal cord tumors likely limits the role of CSF cytology. In this analysis, patients with CSF disease dissemination have worse PSF than others. Therefore, CSF cytology may obviate the need for riskier tissue biopsies; CSF cytology would serve as a method for monitoring tumor recurrence or response to therapy; in cases of STR, recurrence commonly occurs. Scoliosis affects the PSF of patients in this analysis, because complete tumor resection in those patients appears to be more difficult than in others. All of these were not fully discussed in the study of Sun et al.^[7]^

There are some limitations to this study. First, most of the included articles are retrospective studies simply because of the lack of prospective studies in this field. Second, several studies with large samples would be excluded due to unavailable individual data, which may have caused selection bias. Third, the included studies were published in different periods, which could cause heterogeneity of surgical methods and clinical outcomes.

## 5 Conclusion

In conclusion, it is clear that whether it is a TR, adjuvant therapy, chemotherapy, CSF disease dissemination, and scoliosis are influencing factors for PFS: PFS of patients who received TR was much longer than those who received STR. Patients treated with adjuvant therapy or RT and chemotherapy appeared to have shorter PFS than others; PFS of patients with CSF disease dissemination or scoliosis were significantly shorter than others. However, pathological classifications of intramedullary Grade II ependymomas appear not to be associated with PFS. Cellular ependymomas and giant cell ependymomas are associated with OS; cellular ependymomas would have better OS than giant cell ependymoma. However, giant cell ependymoma patients might have the worst OS.

## Acknowledgment

We thank International Science Editing (http://www.internationalscienceediting.com) for editing this manuscript.

## Author contributions

XYS and WW were responsible for designing the search strategy, evaluating the articles, running statistical analysis, and writing this article. CK was responsible for English editing. SBL was responsible for designing the protocol. SYS was responsible for interpreting results. MCG and JZD were responsible for formatting the paper.

**Conceptualization:** Xiang-Yao Sun, Tong-Tong Zhang, Ma-Chao Guo.

**Data curation:** Xiang-Yao Sun, Wei Wang, Tong-Tong Zhang, Chao Kong, Si-Yuan Sun.

**Formal analysis:** Xiang-Yao Sun, Wei Wang, Tong-Tong Zhang, Si-Yuan Sun.

**Funding acquisition:** Shi-Bao Lu.

**Investigation:** Xiang-Yao Sun.

**Methodology:** Xiang-Yao Sun, Ma-Chao Guo, Shi-Bao Lu.

**Project administration:** Xiang-Yao Sun.

**Resources:** Xiang-Yao Sun, Wei Wang, Ma-Chao Guo.

**Software:** Xiang-Yao Sun, Jun-Zhe Ding.

**Supervision:** Xiang-Yao Sun, Tong-Tong Zhang, Jun-Zhe Ding, Shi-Bao Lu.

**Validation:** Xiang-Yao Sun, Tong-Tong Zhang, Jun-Zhe Ding.

**Visualization:** Xiang-Yao Sun, Tong-Tong Zhang.

**Writing – original draft:** Xiang-Yao Sun.

**Writing – review and editing:** Xiang-Yao Sun.

Xiang-Yao Sun orcid: 0000-0001-9385-2402.
